# Ischaemic Meckel’s Diverticulum, Mesodiverticular Band, and Small Bowel Volvulus With Closed Loop Obstruction: A Laparoscopic Approach and Literature Review

**DOI:** 10.7759/cureus.77567

**Published:** 2025-01-16

**Authors:** Chinedu E Unadike, Ayden Ismail, Akshay Harikumar, Omer Ali, Abdul Khan

**Affiliations:** 1 General Surgery, St Mary’s Hospital, Isle of Wight NHS Trust, Newport, GBR; 2 General Surgery, St Mary's hospital, Isle of Wight NHS Trust, Newport, GBR; 3 General Surgery, St Mary's Hospital, Isle of Wight NHS Trust, Newport, GBR; 4 Colorectal Surgery, St Mary's Hospital, Isle of Wight NHS Trust, Newport, GBR

**Keywords:** antimesenteric, diverticulitis, echelon flex, ileoceacal valve, laparoscopic appendicectomy, meckel’s diverticulum, small bowel volvulus

## Abstract

Meckel's diverticulum (MD) is the most common congenital abnormality of the gastrointestinal tract. It usually lies on the antimesenteric side of the ileum, about 60 cm from the ileocecal valve. Histologically, it is a true diverticulum comprising all four layers of the intestinal tract. Complications associated with MD include bleeding, bowel obstruction, intussusception, and inflammation (diverticulitis).

A 12-year-old boy presented to the emergency department with a one-day history of right iliac fossa pain. He had rebound tenderness and localized peritonism. The inflammatory markers were raised. He was listed for an emergency laparoscopic appendicectomy. Intraoperatively, a large necrotic MD was identified, twisted on its pedicle. In addition, a fibrous band extended from the tip of the diverticulum to the posterior aspect of the anterior abdominal wall. A closed-loop, discoloured terminal ileal volvulus with proximal small bowel dilatation was noted. The band was released by sharp dissection, and the terminal ileum volvulus was freed with the return of normal colour and circulation. The gangrenous Meckel’s diverticulum was excised at its pedicle by ECHELON FLEX™ ENDOPATH® staplers (Ethicon, Inc., a Johnson & Johnson company, Raritan, NJ). The postoperative recovery was uneventful.

Early laparoscopic intervention prevented irreversible small bowel ischaemia that may have resulted in resection of the terminal ileum in a child.

## Introduction

Meckel’s diverticulum (MD) arises from an incomplete obliteration of the embryonic vitelline duct, which connects the foetal gut to the yolk sac, typically regressing between the 5^th^ and 7^th^ weeks of gestation. It is the most common congenital anomaly of the gastrointestinal tract, affecting approximately 2% of the population [1]. Complications include bleeding, diverticulitis, obstruction, and perforation, with the risk diminishing with age [1]. The main complication of MD in children is lower gastrointestinal bleeding, and that of adults is small bowel obstruction (SBO) [2]. The mesodiverticular band (MDB), a remnant of the vitelline artery, is a rare complication that increases the likelihood of internal hernias or small bowel volvulus [2]. This case report shows how early laparoscopic intervention averted a complication of ischaemic bowel. The literature review further underscores the benefit of laparoscopic surgery to laparotomy with reference to length of stay. 

## Case presentation

A 12-year-old Caucasian male patient presented to the emergency department with sudden-onset abdominal pain lasting for one day. He had no prior significant medical or surgical history. The pain initially presented centrally and migrated to the right iliac fossa. Accompanying symptoms included one episode each of non-bilious vomiting and diarrhoea, along with chills.

On examination, his vital signs were normal, but his abdomen showed tenderness, guarding, and localised peritonism in the right iliac fossa, with a positive Rovsing's sign. Laboratory results revealed leukocytosis (21.9 × 10^9/L), neutrophilia (18.7 × 10^9/L) and elevated C-reactive protein (9.1 mg/L).

With a clinical diagnosis of acute appendicitis, an emergency diagnostic laparoscopy and appendicectomy was planned without prior imaging. The time between surgical admission to the theatre was approximately four hours. 

**Figure 1:**
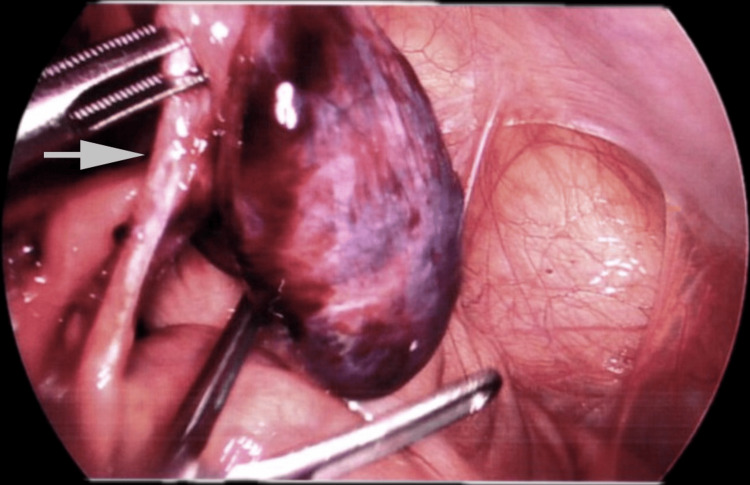
An arrow pointing to the mesodiverticular band suspended with the laparoscopic instrument before the excision of the band

Intraoperatively, an ischaemic MD with an MDB (Figure 1) extending from its tip to the anterior abdominal wall was identified. A 20 cm segment of the small bowel was twisted around the MD, creating a closed loop and proximal bowel dilation. Hemorrhagic fluid was observed in the right paracolic gutter and pelvis.

**Figure 2:**
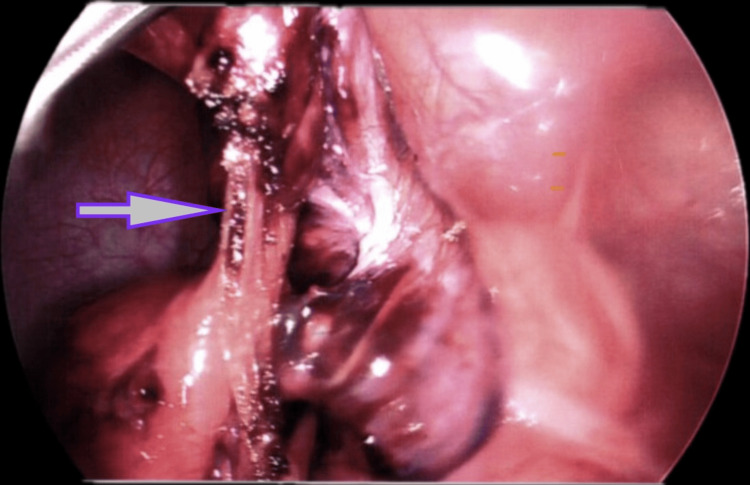
Laparoscopic resection of ischaemic Meckel's diverticulum at the pedicle (the arrow points to the pedicle)

The MDB was divided using diathermy, and the small bowel was untwisted to access the MD. The pedicle of the MD was narrowed with diathermy (Figure 2), and an endostapler (ECHELON FLEX™ ENDOPATH® (Ethicon, Inc., a Johnson & Johnson company, Raritan, NJ)) was used to excise it, ensuring adjacent bowel integrity. Viability of the bruised small bowel was confirmed with warm saline. Haemostasis was achieved, and the MD was retrieved via the umbilical port. Postoperative recovery was uneventful, and the patient was discharged on day four.

**Figure 3:**
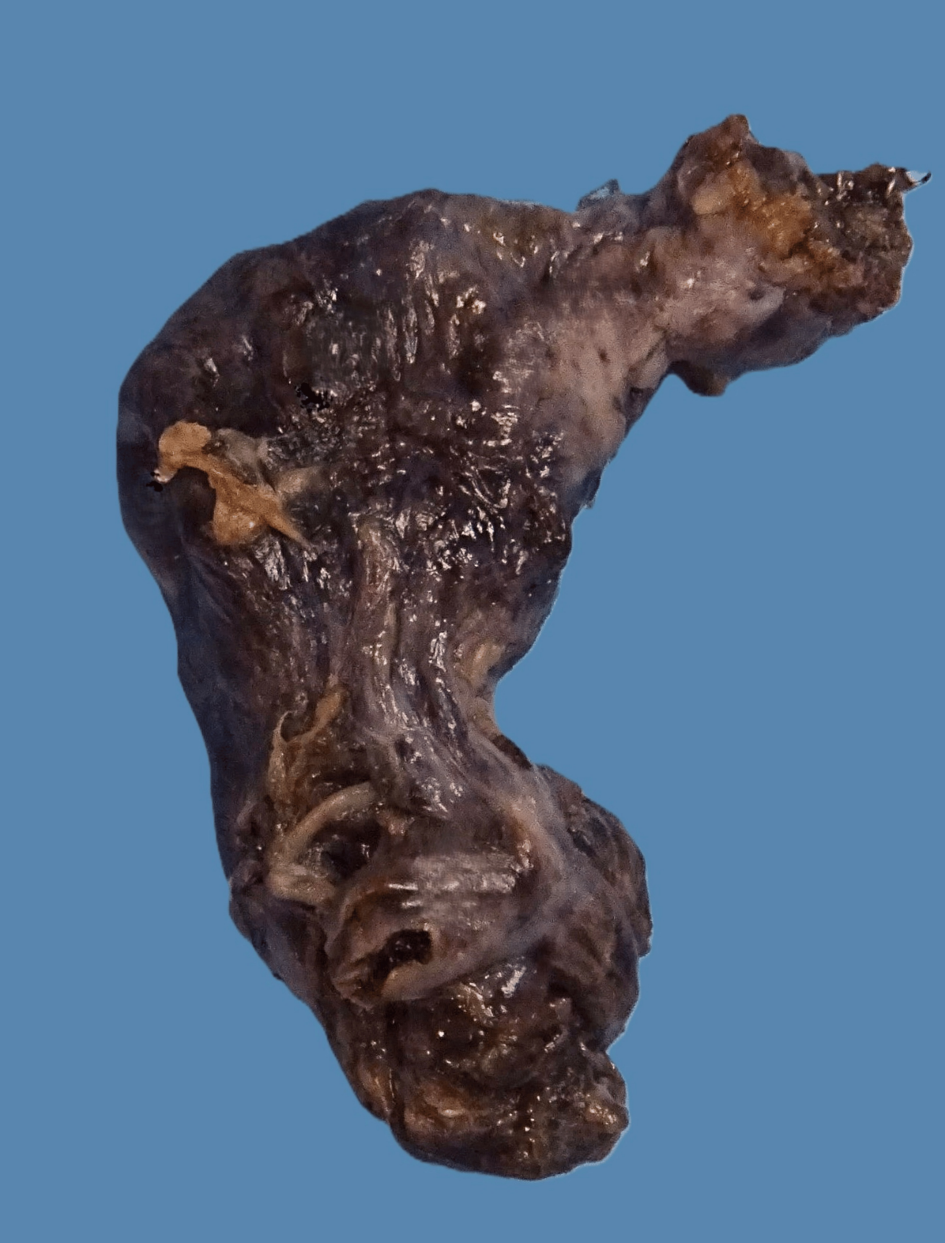
Macroscopic view of Meckel's diverticulum showing congested serosa with diffuse haemorrhagic mottling

**Figure 4:**
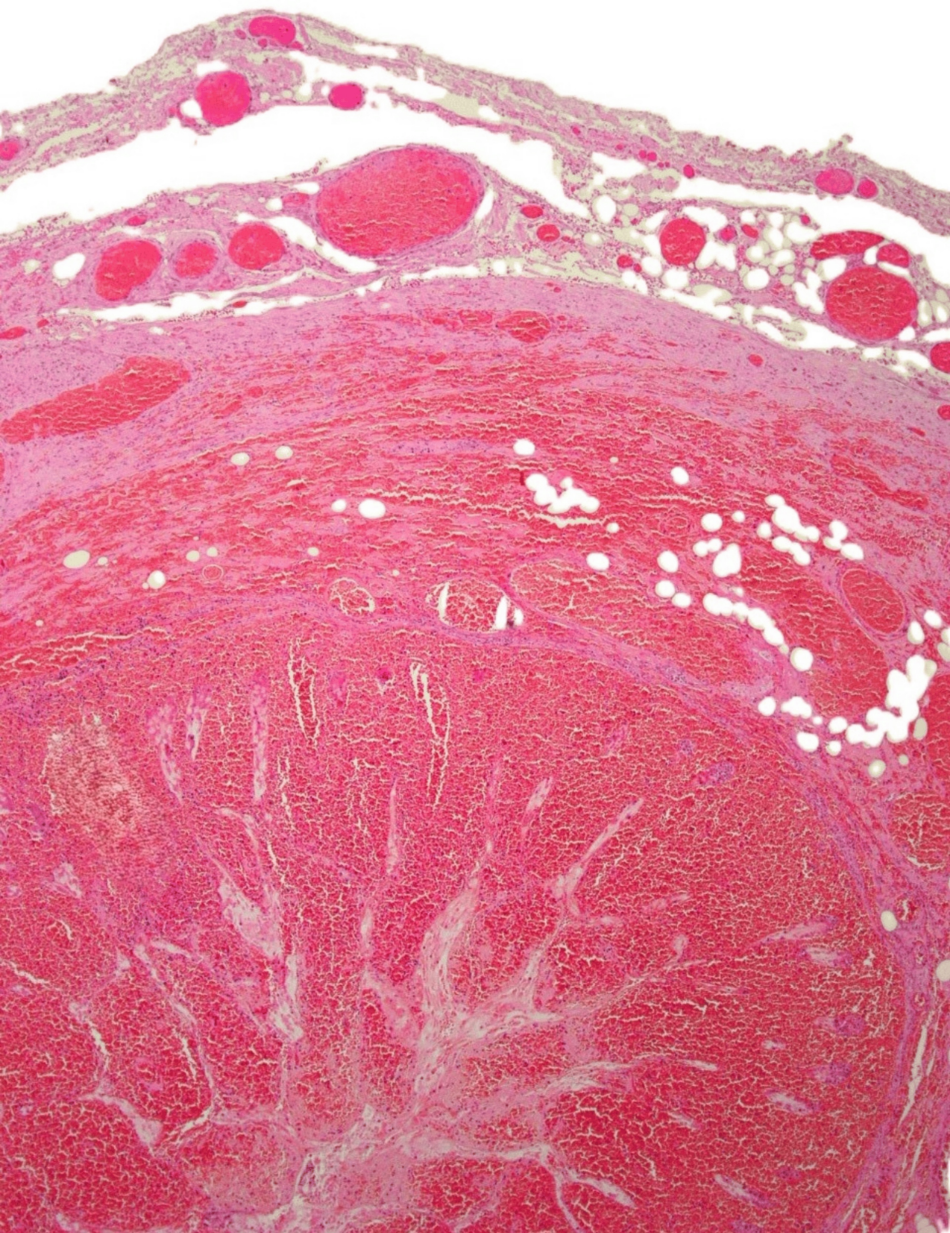
Microscopy showing haemorrhagic infarction of Meckel's diverticulum, with global necrosis of the small bowel mucosa and submucosa. There is also luminal and interstitial haemorrhage.

Histological examination of the MD revealed an infarcted diverticulum (42 × 23 × 15 mm), with congested serosa and diffuse haemorrhagic mottling (Figure 3). The lumen contained clotted blood, and the wall exhibited haemorrhagic infarction without evidence of metaplasia, dysplasia, or malignancy (Figure 4).

## Discussion

Meckel's diverticulum is the most common congenital abnormality of the gastrointestinal tract, affecting 2% of the population. The risk of a lifetime complication from an MD is 4%, with the risk decreasing with age [1]. The complications include bleeding, obstruction, diverticulitis, and perforation [1]. The rare presence of an MDB, a remnant of the vitelline artery, further increases the risk of bowel obstruction and volvulus [3].

The aetiology of bowel obstruction in MD includes intussusception, volvulus, inversion, and herniation through a defect (Littre hernia) [4]. complications related to MDB have been documented across all age groups, with the risk of MDB-related obstruction occurring among adults as well as children [2].

In this case, the MDB resulted in a closed-loop SBO. The decision to proceed with surgery was guided by clinical assessment, despite an initial working diagnosis of acute appendicitis. The patient underwent prompt surgical intervention using a laparoscopic approach, which successfully mitigated the risk of imminent irreversible bowel ischaemia and the need for an emergency small bowel resection.

Laparoscopy has proven to be a valuable diagnostic and therapeutic tool in MD management, with recent literature emphasising the importance of early surgical intervention for MD-related bowel obstruction, including diverticulectomy and, if needed, bowel resection and anastomosis [5].

We identified 16 case reports through the PubMed database, using appropriate Medical Subject Headings (MeSH) terms and Boolean operators to review other cases of MD and MDB causing SBOs from the past five years (Table 1). Two reviewers worked independently to screen free full-text articles in order to avoid bias and add further scrutiny to the literature. For this population, the average age was 36.8 years (range: 0.5-63 years); the majority of patients were reported male (68.8%, 11/16), and the surgical approach for the majority of cases was a laparotomy (75%, 12/16).

**Table 1:** Sixteen studies showed a small bowel obstruction secondary to the mesodiverticular band and the surgical approach. Some cases reported an ischaemic bowel. SBO: small bowel obstruction; LoS: length of stay; M: male; F: female; N/A: not available Age range 20-29 years*, as implied in the original article.

Author	Year	Patient age (years)	Patient's gender	Mesodiverticularband with SBO	Surgical approach	Bowel resection	Ischaemic bowel	Time to surgery (days)	Postoperative LoS (days)	Complications
Zielinski et al. [3]	2023	56	M	Yes	Laparotomy	Yes	Yes	N/A	N/A	None
Skarpas et al. [6]	2020	63	F	Yes	Laparotomy	Yes	No	1	6	None
Chaouch et al. [7]	2023	28	M	Yes	Laparotomy	Yes	Yes	N/A	5	None
Ebrahimi et al. [8]	2021	56	M	Yes	Laparotomy	Yes	Yes	N/A	N/A	N/A
Arslan et al. [9]	2020	63	M	Yes	Laparotomy	Yes	Yes	N/A	7	None
Evan et al. [10]	2024	61	M	Yes	Laparotomy	Yes	No	N/A	7	None
Blum et al. [11]	2024	20-29*	M	Yes	Laparotomy	Yes	Yes	N/A	8	Fever and small intraabdominal fluid collection
Munasinghe et al. [12]	2022	20	M	Yes	Laparotomy	Yes	Yes	N/A	N/A	None
Jha et al. [13]	2021	13	F	Yes	Laparotomy	Yes	Yes	N/A	N/A	None
Soto et al. [14]	2021	6 months	N/A	Yes	Laparotomy	Yes	No	N/A	N/A	None
Kawai et el. [15]	2023	34	F	Yes	Laparotomy	No	No	N/A	7	None
Keot et al. [16]	2023	13	M	Yes	Laparotomy	No	No	2	N/A	None
Alzarea et al. [17]	2022	57	M	Yes	Laparoscopy	Yes	Yes	N/A	3	None
Takura et al. [18]	2021	56	F	Yes	Laparoscopy	Yes	Yes	N/A	5	None
Shimizu et al. [19]	2024	32	M	Yes	Laparoscopy	No	No	5	4	None
Sun et al. [20]	2024	12	M	Yes	Laparoscopy	No	No	N/A	N/A	N/A

Of the cases that utilised a laparotomy, 58.3% (7/12) of patients had an ischaemic bowel, and 83.3% (10/12) underwent bowel resection [3, 6-16]. None of the laparotomy cases that encountered bowel ischaemia reported the time to surgery from hospital admission. Of the laparotomy cases that did not encounter bowel ischaemia, two studies reported their time to surgery as one and two days post admission [6,17]. The average postoperative length of stay for patients who had a laparotomy was 6.7 (five to eight) days.

Of the cases that utilised a laparoscopic approach, ischaemic bowel was encountered in 50% (2/4) of cases, and only these patients with ischaemic bowel underwent bowel resection [17-20]. Laparoscopic surgery for MDB causing SBO without accompanying bowel resection is reported in 12.5% (two out of 16) of total cases [19,20]. Time to surgery from admission was reported in only one article as five days, during which no ischaemic bowel was encountered, nor was bowel resection required [20]. In contrast, this case report details a significantly shorter time from admission to surgery of four hours, with no ischaemic bowel found. For all laparoscopic cases, no complications were reported by the authors. The average postoperative length of stay for patients who were treated with laparoscopy was four (three to five) days, which is consistent with the findings in this case report. 

This case report and literature review highlight the importance of prompt identification of SBO symptoms and proactive management to minimise the risk of bowel ischaemia and consequential bowel resection. Consideration of MD in confirmed SBO is paramount, and emergency exploratory laparoscopic surgery presents a minimally invasive management choice for this pathology. Laparoscopy also provides better positive outcomes and, on average, a shorter postoperative stay compared to laparotomy cases.

## Conclusions

Bowel obstruction secondary to MD and MDB is rare but potentially life-threatening. Timely laparoscopic intervention is crucial in preventing severe complications, such as bowel ischaemia or necrosis. This case underscores the importance of prompt diagnosis and minimally invasive management.

## References

[1] Turgeon DK, Barnett JL (1990). Meckel's diverticulum. Am J Gastroenterol.

[2] Bamarni S, HungFong S, Mino J, Misra S (2021). Systematic review of mesodiverticular band: a rare cause of small bowel strangulation and hemoperitoneum in adults. IJS Short Rep.

[3] Zieliński M, Kaczor P, Jarczyk G, Jackowski M (2023). Small bowel segment with Meckel's diverticulum volvulus related to short mesodiverticular band: a case report. J Med Case Rep.

[4] Chatterjee A, Harmath C, Vendrami CL, Hammond NA, Mittal P, Salem R, Miller FH (2017). Reminiscing on remnants: imaging of Meckel diverticulum and its complications in adults. AJR Am J Roentgenol.

[5] Vargas Aignasse RA, Pantoja Pachajoa DA, Llahi F, Parodi M, Doniquian AM, Viscido GR (2023). Emergency laparoscopic intervention for fibrous band-induced intestinal obstruction and ischemia associated with Meckel's diverticulum: a case report. Int J Surg Case Rep.

[6] Skarpas A, Siaperas P, Zoikas A, Griva E, Kyriazis I, Velimezis G, Karanikas I (2020). Meckel's diverticulitis. A rare cause of small bowel obstruction. J Surg Case Rep.

[7] Chaouch MA, Abdelali M, Hammouda SB, Zayati M, Taieb AH, Noomen F (2023). A case report of small bowel occlusion due to Meckel diverticulum causing a life-threatening condition. Int J Surg Case Rep.

[8] Ebrahimi N, Quinn R, Liang Y, Curran R (2021). A tale of two Meckel’s: small bowel obstruction secondary to Meckel’s diverticulum. J Surg Case Rep.

[9] Arslan HE, Zeren S, Ekici MF, Algın MC (2020). A rare cause of intestinal obstruction: mesodiverticular band. Turk J Colorectal Dis.

[10] Evan C, Christy K, Hanafi RV, Rodjak MW (2024). Acute intestinal obstruction due to Meckel's diverticulum: a case report and literature review. Heliyon.

[11] Blum B, Grimes AD, Carroll HL, Stettler GR (2024). Cecal volvulus secondary to mesodiverticular band. J Surg Case Rep.

[12] Munasinghe BM, Dhanuksha DC, Samarathunga RD, Senevirathne PS, Karunatileke CT (2022). Acute abdomen following axial torsion of a Giant Meckel's diverticulum in a young male: a case report. Int J Surg Case Rep.

[13] Jha SK, Ghimire S, Koirala DP (2021). Torsed gangrenous Meckel's diverticulum causing gangrenous ileal segment: a rare case report of small bowel obstruction in children. Ann Med Surg (Lond).

[14] Urrutia Soto H, Donoso Carrasco C, Carvajal Flores O (2021). Symptomatic Meckel's diverticulum in pediatrics. Andes Pediatr.

[15] Kawai H, Omura N, Hirabayashi T, Shimada T, Kawahara H (2023). Small bowel obstruction due to axial torsion of Meckel’s diverticulum: a case report and literature review. Cureus.

[16] Keot KB, Rajbongshi MC, Medhi R, Barbhuiya EA, Kumar R, Borthakur D (2023). An atypical case of Meckel’s diverticulum with small bowel obstruction: surgical anatomy, embryology and clinical implications. Clin Ter.

[17] Alzarea A, Aljohani A, Qabani H, Alzahrani A, Sairafi R (2022). A rare case of intestinal obstruction caused by Meckel's diverticulum band. Ann Med Surg (Lond).

[18] Takura K, Takayama S, Kani H, Sakamoto M, Ishikawa K, Katada T (2021). A case of intestinal obstruction caused by a mesodiverticular band in Meckel's diverticulum with ectopic pancreas treated by laparoscopic surgery. Int J Surg Case Rep.

[19] Shimizu S, Hara H, Muto Y, Kido T, Miyata R, Itabashi M (2024). Laparoscopic management of small bowel obstruction secondary to a mesodiverticular band of a Meckel's diverticulum in an adult: a case report and literature review. Medicine (Baltimore).

[20] Sun YM, Xin W, Liu YF, Guan ZM, Du HW, Sun NN, Liu YD (2024). Appendicitis combined with Meckel's diverticulum obstruction, perforation, and inflammation in children: three case reports. World J Clin Cases.

